# Serum Albumin Levels: A Biomarker to Be Repurposed in Different Disease Settings in Clinical Practice

**DOI:** 10.3390/jcm12186017

**Published:** 2023-09-17

**Authors:** Elisa Gremese, Dario Bruno, Valentina Varriano, Simone Perniola, Luca Petricca, Gianfranco Ferraccioli

**Affiliations:** 1 Clinical Immunology Unit, Fondazione Policlinico Universitario A. Gemelli-IRCCS, Catholic University of the Sacred Heart, 00168 Roma, Italy; 2 Immunology Core Facility, Fondazione Policlinico Universitario A. Gemelli-IRCCS, 00168 Roma, Italy; 3 Clinical Immunology Unit, Fondazione Policlinico Universitario A. Gemelli-IRCCS, 00168, Roma, Italy; dariobrunomd@gmail.com (D.B.); varrianov@gmail.com (V.V.); perniolasi@hotmail.it (S.P.); 4 Rheumatology Division, Fondazione Policlinico Universitario A. Gemelli-IRCCS, 00168 Roma, Italy; luca.petricca@policlinicogemelli.it; 5 Department of Internal Medicine, Catholic University of the Sacred Heart, 00168 Rome, Italy; gianfranco.ferraccioli@unicatt.it

**Keywords:** albumin levels, pre-albumin levels, emergency department, nephrology, cardiology, oncology, infectious diseases, intensive care units, rheumatology, autoimmune diseases

## Abstract

Serum albumin (ALB), one of the most important proteins in human physiology, has the main functions of maintaining plasma oncotic pressure and plasma volume, transporting hormones, vitamins, oligominerals and drugs, and exerting a powerful antioxidant-anti-inflammatory role. Its prognostic value in liver and malabsorption syndromes is well known. In this narrative review, an analysis of the most important studies evaluating the prognostic significance of low serum ALB levels in hospitalized patients was performed. Specifically, the risk in emergency medicine, cardiovascular diseases, Coronavirus Disease 19 (COVID-19) infection, nephrology, oncology, and autoimmune rheumatic diseases has been examined to fully explore its clinical value. ALB is a negative acute-phase reactant and the reduction in its serum levels represents a threatening parameter for long-term survival in several clinical settings, and a strong biomarker for a poor prognosis in most diseases. Therefore, clinicians should consider serum ALB as a valuable tool to assess the efficacy of specific therapies, both in hospitalized patients and in chronic follow-up.

## 1. Introduction

Albumin (ALB), the most abundant protein in human plasma (about 4.0 g/dL), is synthesized in the liver as a long peptide of 585-aminoacid, with a half-life of approximately 25 days [1]. Due to its remarkable versatility and diverse array of properties, such as binding and transport capability, and osmotic regulation, as well as antioxidant and buffering activity, it has garnered significant scientific attention over the years. Indeed, researchers across various disciplines, including biochemistry, physiology, and medicine, have delved into the intricate roles played by this protein in maintaining homeostasis and regulating essential physiological processes. Thus, considering data on the ALB parameter in the literature, it has a high number of citations in PubMed, being one of the most studied physiological and laboratory parameters in medicine. This narrative review aims to provide an in-depth exploration of the multiple properties of serum ALB, elucidating its profound significance in both health and pathological conditions. Particularly, we have deliberately focused on non-gastroenterological and non-hepatic diseases, to highlight its role in medicine, as a biomarker in many acute and chronic diseases, and to emphasize perspectives on how to obtain the best information in terms of pathophysiology and therapeutic hints in clinical practice.

## 2. Albumin Synthesis and Physiology

The synthesis of human albumin begins in the hepatocyte nucleus, where its genes are transcribed into messenger RNA (mRNA). The mRNA is secreted into the cytoplasm, where it binds to ribosomes that synthesize Pre-Pro-Albumin (Pre-Pro-A) [2]. The Pre-Pro-A has a N-terminal peptide (24 amino acids), which is processed in the lumen of the endoplasmic reticulum, resulting in an extension of 6 still bound amino acids (Pro-Albumin), and releasing the Pre-Albumin. The Pre-Albumin molecule, due to its short half-life (1.9 days) is considered the best parameter for assessing nutritional status and, due to its binding to triiodothyronine (T3) and thyroxine (T4), it is also called transthyretin and thyroxine-binding prealbumin (TBPA). Pro-Albumin is exported into the Golgi apparatus, where the 6 amino acids extension is in turn removed to produce albumin, a single polypeptide containing 585 amino acids.

ALB has a molecular weight of 66.5 kD and is strongly negatively charged. Structurally, it consists of three domains (I, II, III), each of which has two additional subdomains (A, B), containing four and six α-helices, respectively [1].

Only a very small amount of ALB is stored in the liver, and most of it is readily excreted into the blood plasma. Physiologically, 10 to 15 g of ALB are daily released into the bloodstream, accounting for almost half of the total protein content (3.5 to 5 g/dL) of plasma in healthy humans. The synthesis of ALB is stimulated by hormonal factors, such as insulin, cortisol, and growth hormone (GH), while pro-inflammatory mediators, such as interleukin (IL)-6 and tumor necrosis factor (TNF)-α, exert an inhibitory effect. Once produced, only 30–40% of ALB circulates in the blood, while the remaining (70–60%) leaves the vascular compartment at a rate of 5% per hour (transcapillary passage rate) and returns through the lymphatic system; the amount of ALB returning to the vascular compartment is almost equivalent to the amount leaving it. For these reasons, the circulatory half-life of albumin is 16–18 h, while its overall half-life is approximately 3 weeks in healthy young adults. The catabolism of albumin is ubiquitous, mainly taking place at the muscles, liver, and kidneys vascular endothelium level. Serum ALB serves as the primary modulator of fluid distribution throughout the body, being responsible for about 80% of colloidal osmotic pressure (oncotic pressure) [1,2]. This function is exerted both by a direct osmotic effect, related to its molecular weight and high plasma concentration, and by an indirect ability to attract positively charged molecules into the bloodstream, due to its negative charge (*Gibbs–Donnan effect*) [1]. Due to its chemical structure, serum ALB acts as the main carrier of a broad range of endogenous hydrophobic molecules, such as vitamin D, bilirubin, long-chain fatty acids and divalent cations (calcium, magnesium, copper, zinc, thyroxine), and plays a critical role in the delivery of drugs throughout the body [3]. Albumin depletion for any reason [4,5] can lead to extravascular leakage of water and the occurrence of oedema, although a close relationship between albumin level and oedema was not observed in 50 consecutive hospitalized patients in a medical ward in Pretoria, South Africa. In fact, out of 24 patients with ALB levels below 3 gdL, only six had oedema and none of the patients with levels below 1.5 g/dL, had any sign of oedema [6]. This means that other causes should be considered when evaluating the origin of oedema (salt retention from renal disease, cor pulmonale, malignancy, chronic inflammatory diseases, malnutrition, etc.).

Human serum ALB (HSA) is an appealing anti-oxidant molecule [1]. Its antioxidant properties rely on the capacity of binding free redox-active transition metal ions (mainly Cu(II) and Fe(II)) at the N-terminal site and serving as a free radical scavenger [7]. Indeed, these free metal ions may have a high pro-oxidative role through the interaction with hydrogen peroxide (H_2_O_2_), leading to the formation of aggressive Reactive Oxygen Species (ROS) (*Fenton reaction)* [8]. Thus, their binding by HSA limits availability for the Fenton reaction. Moreover, six methionines and 35 cysteine residues are involved in the formation of 17 disulfide bonds [9]. This is based on the reduced sulphydryl groups that can scavenge nitric oxide (NO), hypochlorous acid (HOCL), and other ROS, and on the high affinity site for Cu(II) ions which, together with Fe(II), can be extremely pro-oxidant [10].

The binding of free transition metals limits their availability for the Fenton reaction that catalyzes the production of aggressive ROS [11]. The study of synthetic fractional albumin (and fibrinogen) rates using simultaneous infusions of intravenous [1-14C] leucine and intraduodenal [4,5-3H] leucine, after 22 h of fasting and, during glucose and amino acids absorption, showed an increase in albumin synthesis by 28% [12]. Conversely, inducing a mild acidosis (pH = 7.3) in a normal subject through a 7-day infusion of NH4Cl, resulted in a 20% decrease in ALB synthesis [13]. These studies highlight the dynamics of liver synthetic capacity and suggest that nutrition is certainly important; however, most studies support the idea that albumin is not a good biomarker of nutritional status given its longer half-life [14], while data suggest that pre-albumin levels, due to its shorter half-life, more rapidly reflect the overall level of malnutrition in clinics [15]. A useful algorithm has been proposed to define the pre-albumin status and the risk of poor outcomes (Table 1) [15,16]. Among the hormones that exert a profound effect on the protein synthesis, insulin arose as an important one. Studying diabetic patients’ whole body protein synthesis and fractional synthetic rate of albumin and fibrinogen (using simultaneous 5 h infusions of [14C]leucine and [13C]bicarbonate) during continuous insulin infusion (to maintain euglycemia), and after short-term insulin deprivation, showed that insulin deficiency decreased albumin synthesis by 29%, and increased fibrinogen levels by 50%, increasing whole body proteolysis by 35%.

These dynamic data proved that albumin synthesis (as well as fibrinogen) is an insulin-sensitive process, and that the increase in fibrinogen reflects an acute phase protein response [17]. These data are of fundamental clinical importance.

**Figure 1:**
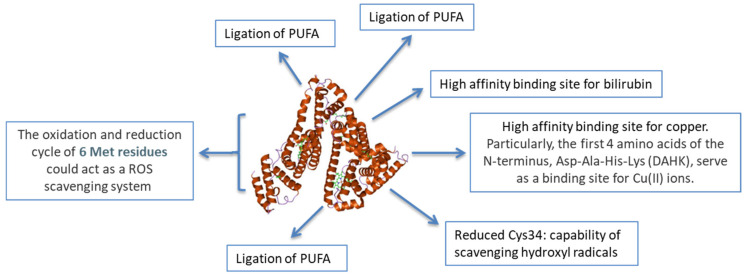
Anti-oxidant properties of Albumin. MET: methionine; ROS: reactive oxygen species; PUFA: Polyunsaturated fatty acids; Cys34: cysteine34; Modified from “The antioxidant properties of serum albumin” [20]. doi: 10.1016/j.febslet.2008.04.057. Epub 2008 May 12. PMID: 18474236.

**Figure 2:**
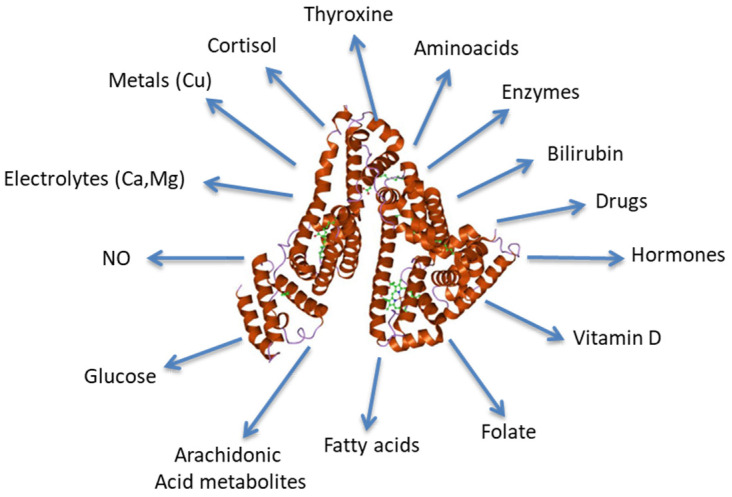
Key substances transported by albumin. NO: nitric oxide. Modified from “The antioxidant properties of serum albumin” [20]. doi: 10.1016/j.febslet.2008.04.057. Epub 2008 May 12. PMID: 18474236.

Albumin exerts several anti-inflammatory effects (Figure 1) and possesses several non-oncotic binding properties (Figure 2) [1,18,19].

## 3. Critical Levels of Albumin in Hospitalized and Intensive Care Unit (ICU) Patients

Hypoalbuminemia, defined as a serum albumin concentration below 3.5 g/dL, is commonly detected in hospitalized adult patients [21], being reported with a prevalence higher than 70% in the elderly [22]. In patients hospitalized for acute illness, albumin emerged as a prognostic factor of death, length of stay, and readmission in 156,511 patients older than 40 years; 21% of patients had albumin < 3.5 g/dL, in-hospital mortality was 14% compared to 4% of those with normal levels (3.5 times higher), and these patients were more likely to stay longer and to be readmitted sooner and more frequently [22].

Hypoalbuminemia can result from a wide range of clinical conditions leading to impaired hepatic synthesis, increased catabolism, or leakage via the gastrointestinal (GI) tract, kidney, skin, or extravascular space, or a combination of these mechanisms [1]. A wide range of clinical conditions may underlie each of these pathophysiological mechanisms. Addressing the first point, liver cirrhosis can result in impaired hepatic ALB synthesis [23], although hypoalbuminemia is clinically evident only in the presence of chronic and severe hepatic insufficiency. Increased catabolism of serum ALB may occur in septic patients, in whom low serum ALB levels result from the synergy of all pathogenetic mechanisms [1]. The third case is typically observed in malabsorption syndromes [24] or in nephrotic syndromes [25]. Moreover, the close connection between serum ALB and inflammation is well recognized. Indeed, inflammatory processes, regardless of the etiopathogenesis, increase capillary permeability, promoting the leakage of serum ALB and, consequently, the expansion of the interstitial space [4,5].

Aside from these typical cases, low serum albumin levels are important predictors of morbidity and mortality even after hospital discharge. Several studies have reported that serum albumin is associated with poor clinical outcomes in severely acutely ill patients. Specifically, it arose as a prognostic factor of morbidity, mortality, and prolonged hospitalization in acutely ill patients [22].

In a meta-analysis, Vincent et al. (2003) reported that each 10 g/L decrease in serum ALB levels is related to an increase in the odds of mortality by 137%, morbidity by 89%, and prolonged ICU stay by 28%, respectively, regardless of nutritional status [21].

Interestingly, analysis of dose dependence in controlled trials of albumin therapy indicated that the complication rate may be lower if serum ALB concentration exceeds 30 g/L during ALB supplementation [26].

Moreover, Eckart et al. (2019) prospectively investigated the association between nutritional status, inflammation, and low serum ALB levels (<3.4 g/dL) and 30-day mortality in a cohort of 2465 patients in the emergency department of a Swiss tertiary care center, finding that hypoalbuminemia correlated with systemic inflammation and high nutritional risk, and independently predicted 30-day mortality (Odds Ratio (OR): 2.87, 95% Confidence Interval (CI):1.70–4.84, *p* < 0.001) [27].

All these data suggest that albumin levels in the emergency department can already be considered a strong biomarker of an overall outcome in the hospitalized patients. At the same time, inflammation must be determined in various disease states [28,29,30]. In fact, TNF has shown effects on acute phase proteins on its own [31], IL-1α has been shown to stimulate hepatic synthesis of some proteins up to 1000-fold (serum amyloid A-SAA), and 2–10 fold of fibrinogen, complement components, factor B, metallothionins, but also to decrease the transcription of RNA encoding albumin (as well as transferrin, liprotein lipase, and cytochromes) [32]. In primary cultures of human hepatocytes, it has been shown that IL-6 induces C-reactive protein (CRP) and SAA in a dose-dependent manner, as well as fibrinogen, α1-acid glycoprotein, α2 macroglobulin, haptoglobin, and to decrease albumin and pre-albumin expression [33]. The strongest downregulation of the albumin gene and the increase in CRP were observed with the combination of IL1α and IL6 [34].

Based on the above, serum ALB concentration appears to be a reliable tool for mortality risk stratification in the context of a medical emergency.

## 4. Albumin and Cardiovascular Disease

Conceptually, serum ALB levels can have a massive impact on cardiovascular (CV) pathophysiology, due to its oncotic, antioxidant, anti-inflammatory, and anticoagulant/antiplatelet aggregation actions.

Although the prevalence of hypoalbuminemia in CV diseases has not been precisely elucidated, its frequency has been reported to be consistent and extremely varied in several meta-analyses addressing deleterious conditions such as heart failure (HF), coronary artery disease (CAD), atrial fibrillation (AF), cerebrovascular accident (CVA) and peripheral artery disease (PAD), ranging from nearly 13% in stable CAD to 90% in elderly patients with severe HF [35,36].

Interestingly, in this setting of CV diseases, low serum ALB levels have arisen as a robust poor prognostic factor independent of the most common modifiable and non-modifiable risk factors and confounders, including systemic inflammation and malnutrition, thus suggesting a causative role of other physiological functions of serum ALB [35,36,37].

In particular, in the Copenhagen General population study (100,520 subjects with a follow-up of 8.5 years), it emerged that for each 1 g/dL lower albumin level (hypoalbuminemia defined as <3.5 g/dL), the Hazard Ratios (HRs) were 1.17 for ischemic heart disease, 1.25 for myocardial infarction, 1.37 for any stroke, and 1.46 for ischemic stroke [35].

Specifically, hypoalbuminemia has been shown to be common in heart failure patients, emerging as a reliable tool to identify patients at high risk of in-hospital and long-term mortality, with a similar predictive value to that reported for serum brain natriuretic peptide (BNP) [38,39].

An analysis of 576 consecutive patients with HF and preserved ejection fraction (HFPEF), hospitalized and followed-up for 30 days, revealed hypoalbuminemia (<3.4 gr/dL) in 160 (28%) patients. After 30 days, CV mortality was 21.8% in hypoalbuminemic patients, with respect to 8.9% in normoalbuminemic patients (2.44-fold higher risk, *p* < 0.001) [40]. Renal dysfunction has been considered the pathophysiological mechanism leading to hypoalbuminemia.

Data from a prospective cohort study enrolling 734 patients with stable coronary artery disease stratified by baseline serum ALB concentration in the low serum albumin group (<3.5 g/dL, *n* = 98) and in the normal albumin group (≥3.5 g/dL, *n* = 636), demonstrated that lower baseline serum albumin was related to an increased risk of all-cause mortality (10.2 vs. 0.5%, *p* < 0.001) and serious CV events (7.1 vs. 1.4%, *p* < 0.001) regardless of comorbidities and demographic characteristics (all-cause mortality, HR 6.81, 95% CI 1.01–45.62; hard CV events, HR 3.68, 95% CI 1.03–13.19) [41]. Moreover, when analyzing 1303 patients with acute coronary syndrome (patients with ST-segment elevation myocardial infarction (STEMI), non-STEMI (NSTEMI)), and unstable angina, undergoing coronary angiography, a baseline albumin level <3.65 mg/dL, elevated systolic blood pressure, and a high SYNTAX score [42], which is a widely used composite score to assess the complexity and severity of CAD, emerged as independent predictors of in-hospital mortality. Interestingly, albumin level per se was strongly predictive of the SYNTAX score [42].

Concerning cardiac arrhythmias, serum albumin has been shown to impact the electrical function of the myocardium [43], although there are few data assessing its correlation and cardiac rhythm abnormalities. A retrospective case–control study in China, aimed at evaluating the clinical features of 950 patients suffering from atrial fibrillation and 963 age-and sex-matched non-AF individuals with sinus rhythm, reported a higher incidence of low serum ALB concentration in adults men who experienced AF, regardless of potential confounding covariates [44].

There are still limited long-term studies focusing on the association between hypoalbuminemia and cerebrovascular accident. According to the data from the Third China National Stroke Registry (CNSR-III), low baseline serum ALB levels (<3.5 g/dL) arose as a risk factor associated with poorer outcomes and mortality in acute ischemic stroke (AIS) or transient ischemic attack (TIA) patients at 3-month follow-up [45].

Interesting data on peripheral artery disease and CV outcomes have been published. In particular, Chahrour MA et al. (2021) retrospectively explored data from 35,383 patients undergoing lower limb amputation to investigate the relationship between preoperative hypoalbuminemia and postoperative mortality; the authors concluded that the mortality rate was higher in patients with very low serum albumin concentration (<2.5 g/dL) compared to low (2.5–3.39 g/dL) and normal levels (≥3.4 g/dL) (11%, 6.8%, and 3.9%, respectively), even after adjusting for confounding variables [46]. As far as the drugs most commonly used in the setting of CV diseases, severe hypoalbuminemia has been shown experimentally to be involved in the mechanisms of resistance to diuretics. Although it has not yet been satisfactorily interpreted in the clinical setting, the role of human serum ALB as a major drug carrier appears to be an additional weapon to influence the etiopathogenesis of CV disease [47]. Additionally, the observation that statins stimulate ALB synthesis by cultured Hep2 cells [48], emphasize once more the comprehensive anti-inflammatory effects on ALB exerted by these drugs [49].

Of further interest, the combination of statin and metformin has been shown to attenuate the diabetic cardiomyopathy by dampening oxidative stress, apoptosis and inflammation in type 2 diabetic mice [50].

All mentioned data promote serum ALB levels as a potential modifiable risk factor, as well as a powerful prognostic marker in the context of CV diseases. Although the clinical efficacy of ALB supplementation is still debated, it is conceivable to consider the treatment of all possible underlying conditions leading to hypoalbuminemia as a huge priority in the management of CV diseases.

## 5. Albumin and COVID-19

Research encompassing biomarkers for predicting COVID-19 patient outcomes is rapidly increasing, and higher albumin levels upon admission have emerged as predictors of better prognosis in hospitalized patients with confirmed COVID-19 infection. Biologically, ALB has been shown to exert a down-regulating effect on the production of angiotensin converting enzyme 2 (ACE2), which is the target receptor for COVID-19 [51].

Moreover, glycated-ALB serves as a high affinity binding protein for SARS-CoV-2 spike proteins, and this may contribute to immune evasion and influence the severity and the pathology of SARS-CoV-2 infection, especially in prediabetes and diabetes [52].

Prior to the COVID-19 pandemic, hypoalbuminemia was already considered a poor prognostic factor in patients hospitalized for community-acquired pneumonia (CAP) [53], significantly affecting the 30-day mortality rate [54,55].

Hypoalbuminemia, which is suggestive of malnutrition, appears to profoundly impact outcomes in COVID-19 infected patients regardless of age and morbidity, being related to a greater prevalence of cardiac injury and hypercoagulability, thereby increasing the burden of inflammatory disease [56]. Moreover, the prevalence of malnutrition in hospitalized COVID-19 ranges from 14 to 70%, extending the length of hospitalization and leading to disability and reduced quality of life after hospital discharge [57].

Of interest, Violi F et al. (2021) provided evidence that ALB supplementation reduces hypercoagulability in hospitalized patients with laboratory-confirmed COVID-19 and SARS-CoV-2-related pneumonia in a small Italian cohort [58].

Furthermore, Arnau-Barres I et al. (2021) evaluated the impact of serum ALB levels on COVID-19-related in-hospital mortality adjusted for potential confounders, reporting that deceased patients were older, had more comorbidities, higher inflammatory status, and lower serum albumin levels at time of hospitalization (3.10 g/dL (0.51) vs. 3.45 g/dL (0.45); *p* < 0.01). Interestingly, severe hypoalbuminemia (<3 g/dL) at baseline strongly predicted in-hospital mortality in a multivariate logistic regression model controlled for age, inflammation, comorbidities, and severity at admission (OR 2.18 95% CI 1.03–4.62; *p* = 0.039) [59].

By transferring these data to the emergency department, Turcato et al. (2022) observed that, in patients infected with COVID-19, serum ALB levels <3.5 g/dL were independently associated with the presence of severe infection and 30-day mortality [60].

Beyond the serum ALB level itself, recent studies have also investigated the role of clinical indices that include albumin as a variable. Specifically, the CRP/albumin ratio (CAR) arose as an independent risk factor for 30-day mortality rate in patients with COVID-19 [61].

More recently, the ferritin/albumin ratio (FAR) has been identified as a promising tool for predicting 28-day mortality in patients treated for severe COVID-19 pneumonia in an ICU setting [62].

Still in an ICU setting, Ertekin B et al. (2023) retrospectively analyzed a cohort of 619 patients suffering from severe COVID-19 disease, investigating the mortality rate with regard to serum ALB levels, blood urea nitrogen (BUN)/ALB ratio (BAR), D-dimer/ALB ratio (DAR), C-reactive protein (CRP)/ALB ratio (CAR), and neutrophil/ALB ratio (NAR). Interestingly, all the studied ratios were found to be detrimental risk factors for mortality, being more valuable than serum ALB level alone in predicting prognosis [63].

Similarly, a urea/ALB ratio (UAR) ≥ 12.17 was observed to double the risk of ICU mortality in hospitalized COVID-19 patients, regardless of other inflammatory parameters [64].

Finally, Jovicic BP et al. (2023) demonstrated the interrelationships between serum ALB concentration, vitamin D, and D-dimer in patients diagnosed with COVID-19, as well as their significance as predictors of the need for supplemental oxygen [65].

Thus, based on the above, it is conceivable that serum ALB could serve as a reliable tool to detect in-hospital complications in COVID-19 disease.

## 6. Albumin in Nephrology

Serum ALB serves as an established and risk-adjusted predictor of progression to end-stage renal disease (ESRD) and all-cause mortality in patients with chronic kidney disease (CKD), especially in more vulnerable populations, such as HIV-infected and elderly adults [66].

In this context, hypoalbuminemia mainly occurs as a manifestation of protein-energy wasting, which is a state of metabolic and nutritional alterations characterized by loss of protein and energy stores leading to cachexia [67]. Of particular interest, in diabetes and in diabetic nephropathy, metformin has been shown to induce the synthesis of ALB by primary cultured hepatocytes and to reduce albuminuria and exert a profound anti-inflammatory renoprotective role in diabetic nephropathy in vivo. [68]. The anti-inflammatory effect of metformin irrespective of diabetes status emphasizes the crucial role that the coexistent anti-inflammatory pharmacologic effects on ALB may produce by drugs primarily employed for their specific mechanism of action [69].

Notably, hypoalbuminemia represents an established risk factor for infection-related in-hospital death in patients with end-stage renal disease undergoing hemodyalisis [70].

Additionally, ESRD patients undergoing hemodialysis face an even greater risk of hypoalbuminemia due to ALB losses during dialysis [71].

Admur RL et al. (2019) demonstrated that in the context of chronic kidney disease, baseline serum ALB level can act as a valid tool to predict the occurrence of atherosclerotic vascular disease beyond the conventional assessment of cardiovascular risk factors [72].

Kawai et al. (2018) pictorially reported the risk-adjusted correlation between low baseline serum ALB concentration and worsening renal outcome in patients suffering from Immunoglobulin A (IgA) nephropathy [73], which is the most common type of idiopathic glomerulonephritis worldwide [74].

Remarkably, oxidative stress on mesangial cells has been shown to be a key pathogenic factor in the onset and progression of IgA nephropathy [73], thus suggesting that the protective action of serum ALB in this context mostly relies on its massive antioxidant capability [75].

Additionally, in primary membranous nephropathy, patients who have achieved partial remission (proteinuria <3.5 g/day and relative reduction ≥50% with preserved glomerular filtration rate) with serum ALB levels >3.5 g/dL showed a lower risk of relapsing disease if compared than those with lower serum ALB concentration [76].

In conclusion, data suggest that serum ALB could be a feasible tool for early identification of patients at higher risk, regardless of the ehtiopatogenesis of renal damage. However, in such a range of plausible mechanisms leading to hypoalbuminemia in CKD patients (i.e., proteinuria, chronic inflammation, inappropriate nutrition), further studies are needed to detect in which type of patients the ALB supplementation may be more beneficial [77].

## 7. Albumin in Oncology

Malnutrition and cachexia in cancer patients are major challenges, negatively impacting response to treatment and overall quality of life [78]. Among the broad spectrum of methods to assess nutritional status in cancer, serum ALB levels have emerged as one of the most accurate [79].

Nevertheless, serum ALB is not only a tool to investigate the nutritional status of cancer patients: in the hospital setting, it is well-known that serum ALB is contingent upon length of stay and all-cause mortality in cancer patients, regardless of type of malignancy, considered alone or in combination with other factors [80].

Notably, while serum ALB concentration usually remains in the normal range in the early stages of cancer, it has been reported to dramatically decrease as the disease progresses, thereby serving as a valuable prognostic marker.

The modified Glasgow Prognostic Score (mGPS), a widely used composite score including CRP and serum ALB to estimate postoperative outcome in cancer patients, has been shown to be a predictor of mortality among patients undergoing chemotherapy for unresectable colorectal cancer [81].

Choi et al. (2008) investigated the risk factors of early recurrence after curative resection of hepatocellular carcinoma, reporting that serum ALB level less than or equal to 3.5 g/dL at time of recurrence is a poor prognostic factor for overall survival at 1, 3, and 5 years [82].

An elegant study prospectively evaluating 3-year survival in localized non-small cell lung cancer reported serum ALB levels among factors associated with the worst outcome [83].

For female cancers, in a cohort of 213 histologically confirmed cases of ovarian cancer, serum ALB levels ≥3.6 g/dL were associated with a median survival of 23.3 months (95% CI, 16.5–30.1 months), compared to a median survival of 7.3 months (95% CI, 4.8–9.8 months) in those with low serum ALB levels, regardless of disease stage, treatment, and serum cancer antigen-125 [84].

Moreover, low preoperative serum ALB levels (<4.0 g/dL) were associated with shorter recurrence-free survival and overall survival in a cohort of 157 patients who underwent surgery for breast cancer, irrespective of canonical poor prognostic factors, including histological and receptor expression [85].

Recently, Yoo SK et al. (2022) reported elevated pretreatment serum ALB concentrations to favorably predict radiographic response to immune checkpoint blockade (ICB) among 16 cancer types, proposing baseline serum ALB as a valuable biomarker for determining ICB outcome and patients prognosis along with genomic factors [86]. Of clinical interest, given the possible comorbidities, metformin provided a beneficial effect on head and neck cancers in terms of overall survival [87], again showing that the anti-inflammatory effects can be present in various clinical settings.

In conclusion, in the era of transcriptomic, proteomic, and metabolomic analyses towards precision medicine in oncology, serum ALB might arise as costless and readily available predictor of cancer progression and survival.

## 8. Albumin in Rheumatologic Autoimmune Diseases

The well-known antioxidant capacity of serum ALB is pivotal in such a range of immune-regulatory mechanisms. Since the majority of immune-mediated inflammatory diseases (IMIDs) are characterized by a down-regulation of serum ALB in response to the inflammatory milieu [88], it is conceivable that its assessment could arise as a useful marker for monitoring the ongoing activity as well as response to treatment.

Decreased serum ALB levels have been extensively described in rheumatoid arthritis (RA), mainly due to suppression of hepatic production by inflammatory cytokines storm, hemodilution and malnutrition status [89].

Ganeb S et al. (2020) observed that serum ALB levels are significantly lower in RA subjects (median 3.9; 3.5–4.35; *p* < 0.001) than in healthy controls; moreover, when stratifying RA patients by disease activity, as assessed by Disease Activity Score-28 (DAS28), serum ALB was significantly decreased in patients with high disease activity compared with both those in moderate activity and those in remission [89].

Additionally, a case–control study in Japan described low serum ALB levels (≤4.2 g/dL) to be independently associated with the prevalence of osteoporosis in a cohort of 197 postmenopausal women with RA [90].

Hypoalbuminemia has been frequently reported in systemic lupus erythematosus (SLE) [91,92,93]. In this context, apart from the canonical underlying pathogenic mechanisms, nephritis may account for nephrotic range proteinuria and, consequently, low serum ALB levels.

In a cohort of 1078 patients diagnosed with SLE, serum ALB concentration inversely correlated to overall SLE disease activity, especially in those suffering from lupus nephritis and proteinuria [94].

Furthermore, data from the NYU Specimen and Matched Phenotype Linked Evaluation (SAMPLE) Lupus Registry demonstrated that, in lupus nephritis, serum ALB levels >3.7 g/dL at 12 months of follow-up after renal biopsy predicted good long-term renal outcome [95].

Recently, Ahn SS et al. (2022) prospectively evaluated the relationship between disease activity and serum ALB, prealbumin, and ischemia-modified albumin in patients with anti-neutrophil cytoplasmic antibody (ANCA)-associated vasculitis (AAV), revealing that only serum ALB concentrations increased consensually when disease activity improved [96].

All this evidence supports the possibility of including serum ALB measurement in such a composite score range to better and easily stratify patients and improve their outcomes in rheumatic diseases.

## 9. Conclusions and Future Directions

Due to the aforementioned physiological functions, a robust correlation between serum ALB levels and general health results understandable. The levels of ALB depend on gene function: to date, 77 of its mutations are known, of which 65 result in bis-albuminemia, and five lead to analbuminemia (<1 g/L) [97]. These are very rare, thus supporting ALB levels as a strong indicator of normal physiology in the clinical practice [1].

Low serum ALB levels have emerged as a detrimental factor in a broad range of clinical settings, serving as an independent baseline predictor of poor outcomes (Table 2). Although there is no clear evidence on the benefits of ALB supplementation, nor stringent indications to assess serum levels during the follow-up of chronic diseases, it is evident that any treatment capable of preserving the intravascular ALB pool could improve the prognosis of patients, particularly when performed at an early stage.

Regarding future perspectives, the ischemia-modified albumin (IMA) is a variant of ALB characterized by an altered structural conformation of the N-terminus, which mainly occurs during prolonged tissue ischemia and inflammation [98].

This variant has a lower binding capacity to cobalt, and studies suggest that it may sensitively reflect myocardial ischemia (it can be measured by an albumin-cobalt binding assay). Moreover, recent studies report IMA as a valid biomarker of collateral vessels formation in coronary myocardial disease [99].

Of interest, IMA has also been observed to be upregulated in patients with autoimmune disorders including ankylosing spondylitis, Behcet’s disease, and inflammatory bowel diseases [100,101,102].

Furthermore, oxidative forms of human ALB (human mercapto-albumin (HMA), human non-mercapto-albumin (HNA)) appear to be promising biomarkers of oxidative stress. Although previous studies have demonstrated the upregulation of HNA in a variety of chronic conditions such as CKD, diabetes mellitus, and liver cirrhosis [103,104,105], there are no published data investigating it in the context of rheumatic diseases. Considering the pivotal role of oxidative stress in the pathogenesis of atherosclerosis and the well-known increased risk of CVD in patients suffering from rheumatic diseases, oxidative forms of human ALB could help clinicians earlier identify patients in whom a more stringent follow-up is needed.

## Figures and Tables

**Table 1:** Algorithm to define the nutritional status and the risk of poor outcome [15].

Prealbumin Levels	Risk
15.0 to 35.0 mg/dL	Normality
11.0 to 15.0 mg/dL	Below the normality, increased risk
5.0–10.9 mg/dL	Malnutrition, severe risk
<5.0 mg/dL	Poor prognosis

**Table 2:** Albumin levels raising concerns in hospitalized patients in various clinical settings.

Clinical Setting	ALB Levels	Risk	Reference
Emergency Department (ED)	<3.4 g/dL	30 days mortality, OR: 2.87	[27]
Hospital Discharge	<3.4 g/dL	3 months mortality, 2.5 fold higher	[22]
CKD hospitalized	<2.5 g/dL	Mortality, 4.9 fold higher risk	[70]
Diffuse large B cell lymphoma	<3.5 g/dL	In-hospital death, 2.0 fold higher	[80]
HFPEF hospitalized	<3.4 g/dL	30 days death, 2.4 fold higher risk	[40]
CAP hospitalized	<3.0 g/dL	30 days mortality, OR: 2.11	[55]
COVID-19 pneumonitis at ED	<3.5 g/dL	30 days mortality, OR: 2.92	[60]
CICU (Cardiologic-ICU)	<3.4 g/dL	In-hospital mortality, 2.3 fold higher	[27]
Nephritic SLE	>3.7 g/dL	At 1 year follow-up post biopsy, HR 0.14	[95]

ALB: albumin; CKD: chronic kidney disease; HFPEF: heart failure with preserved ejection fraction; CAP: community acquired pneumonia; CICU: cardiologic patients in ICU; OR: odds ratio; HR: hazard ratio; SLE: systemic lupus erythematosus.

## Data Availability

Not applicable.
